# Navigating protein – nucleic acid sequence-structure landscapes with deep learning

**DOI:** 10.1016/j.sbi.2025.103162

**Published:** 2025-09-22

**Authors:** Elodie Laine, Sergei Grudinin, Roman Klypa, Isaure Chauvot de Beauchêne

**Affiliations:** 1 Department of Computational, Quantitative, and Synthetic Biology (CQSB), UMR 7238, IBPS, Sorbonne Université, CNRS, Paris, 75005, France; 2 Institut Universitaire de France (IUF), France; 3 Univ. Grenoble Alpes, CNRS, Grenoble INP, LJK, Grenoble, 38000, France; 4 LORIA, CNRS, Inria, University of Lorraine, Vandoeuvre-lès-Nancy, 54506, France

**Keywords:** Protein-NA complex, RNA design, Generative modeling, Deep learning

## Abstract

A few years after AlphaFold revolutionised the field of protein structure prediction, the new frontiers and limitations in structural biology have become clearer. Predicting protein–nucleic acid interactions currently stands as one of the major unresolved challenges in the field. This knowledge gap stems from the scarcity and limited diversity of experimental data, as well as the unique geometric, physicochemical, and evolutionary properties of nucleic acids. Despite these challenges, innovative ideas and promising methodological developments have emerged for both predicting protein–nucleic acid complex structures and designing nucleic acids capable of binding to specific protein conformations. This review presents these recent advances and discusses promising avenues, including the integration of high-throughput profiling data, the development of more rigourous and richer evaluation benchmarks, and the discovery of biologically meaningful regulatory and structural signals using self-supervised learning.

## Introduction

Interactions between proteins and deoxyribo(D-) or ribo (R-) nucleic acid (NA) molecules play essential roles in a plethora of biological processes, including genome replication and protection, gene expression, transcription and splicing, protein translation, and the immune response, among others. Moreover, protein–RNA interaction networks have recently emerged as promising therapeutic targets, due to the numerous diseases associated with their impairment – cancer, cardiovascular disease, and neurodegenerative disorders, and the explosion of high-throughput biochemical methods for profiling these interactions [1]. Hence, deciphering the recognition mechanisms and binding modes underlying protein–NA interactions is essential for fundamental biology as well as medicine. Nevertheless, our knowledge about protein-NA complexes has been lagging far behind that of protein–protein complexes.

Despite having greatly increased in the last decades, the number of protein–NA complex structures available in the Protein Data Bank (PDB) [2,3], around 14,750 as of June 2025, is still dramatically smaller compared to that of proteins and homomeric protein complexes [2]. Moreover, the set of known protein–NA complexes lacks diversity, with the ~6,500 experimentally resolved protein–RNA complexes encompassing only a few short and highly folded RNA families, such as tRNAs, riboswitches and ribozymes [4,5]. This difficulty in experimentally solving protein–NA complex structures calls for the development of high-throughput and accurate predictive methods.

In this review, we critically assess deep learning methods for predicting protein-NA complex structures, highlighting specific properties of nucleic acids that may limit current accuracy. We also examine the related challenge of designing nucleic acids that bind to target proteins, and explore promising future directions. Our focus is primarily on RNA, reflecting both the greater research attention it has received and its central role in many regulatory and therapeutic applications.

### Deep learning has not yet revolutionised protein–NA complex prediction

**Figure 1:**
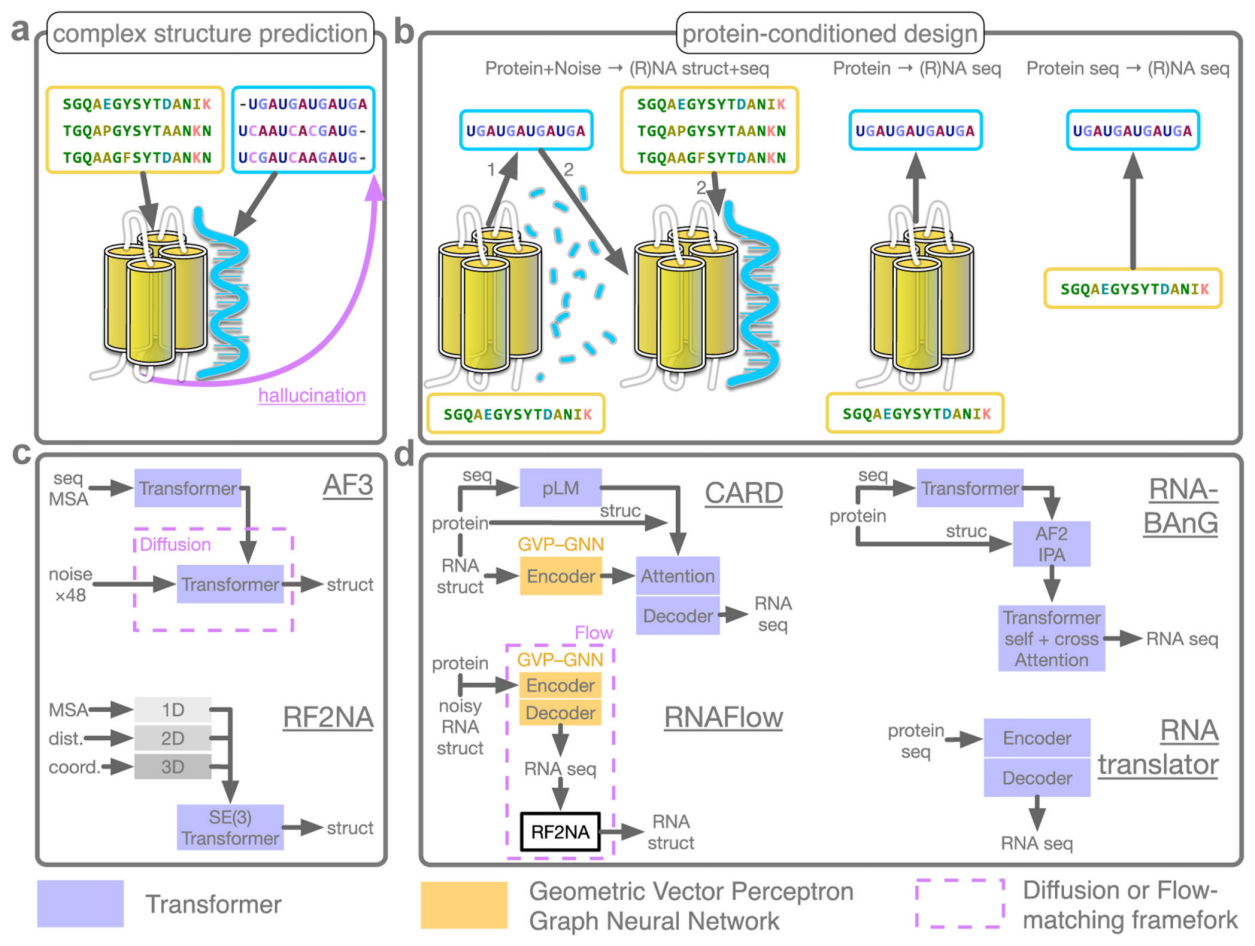
Overview of the protein–NA prediction paradigms discussed in the review. (**a**) Prediction of protein-NA complex structures starting from multiple sequence alignments. The purple arrow indicates NA sequence design through hallucination (*e*.*g*., BindEnergyCraft [25]). (**b**) Design of NA competent for binding a given protein. Left: co-design of NA sequence and structure starting from a fixed protein conformation and a noisy RNA structure. Middle: design of NA sequence conditioned on an input protein conformation. Right: design of NA sequence conditioned on a protein sequence. The experimental structural data contained in the PDB can be augmented with omics data, for instance coming from SELEX experiments, to improve training. (**c**)**-**(**d**) Diagrams depicting some deep neural network architectures designed for these tasks. (**c**) AF3 [10] and RF2NA [8] for structure predictions (**a**). (**d**) CARD [26], RNAFlow [27], RNA-BAnG [28], and RNAtranslator [29] for protein-conditioned RNA design (**b**).

The spectacular advances in protein structure prediction [6,7] have stimulated a strong interest in expanding successful deep learning architectures to model nucleic acids in addition to proteins (Figure 1a,c and Table 1). RoseTTAFoldNA (RFNA) emerged as the first deep learning method specifically designed for protein-NA complex prediction [8], with a 3-track neural network operating on protein and NA multiple sequence alignments (MSA), geometry, and 3D coordinates, stacked with an SE(3)-equivariant transformer [9] for refinement. It was quickly followed by AlphaFold3 (AF3) [10] whose advances compared to AlphaFold2, beyond handling nucleic acids and other molecules, include simplifying the treatment of sequence information, introducing a denoising diffusion framework for refining the 3D coordinates, favouring data augmentation over strict SE(3) equivariance, and enriching the training data with AF-Multimer distillation. Several open-source adaptations of AF3 are now available, such as the Boltz series [11] and HelixFold3 [12].

However, these generalised models for predicting bio-molecular complexes have not yet met the scientific community’s expectations. Indeed, the latest edition of the Critical Assessment of Techniques for Protein Structure Prediction (CASP16) emphasised limitations in deep learning-based methods for protein-NA interaction structure prediction, which fail to outperform more traditional approaches without human expertise [13]. The AF3 server [10] was ranked 16th and 13th (lDDT and i-lDDT) overall for protein-NA interface and hybrid complex prediction in CASP16. All deep learning predictors performing better than this baseline either directly used or adapted AF and RFNA architectures, achieving enhanced performance through expert manual intervention, deeper sequence search combined with Language Model (LM) embeddings, better template identification, and refinement with classical docking or molecular dynamics simulations [14] (Table 1). Nonetheless, none identified residues involved in the interface for the two targets that lacked templates in the PDB, highlighting that protein-NA complex structure prediction still largely relies on the availability of homologous experimental structures as templates [13]. Focusing on protein-RNA complexes, the authors of AlphaFold3 reported a success rate of only 38 % for a small test set of 25 complexes with low homology to known template structures, compared to 19 % for RoseTTAFold2NA [10]. A comprehensive bench-marking study of the two predictors on over a hundred protein-RNA complexes further confirmed these results: AF3 outperforms RF2NA but its predictive accuracy remains modest, with an average TM-score of 0.381 [15]. AF3 struggles in modelling protein-RNA complexes beyond its training set and in capturing non-canonical contacts and cooperative interactions [5].

### Nucleic acids are not proteins: the challenge of flexibility

**Figure 2:**
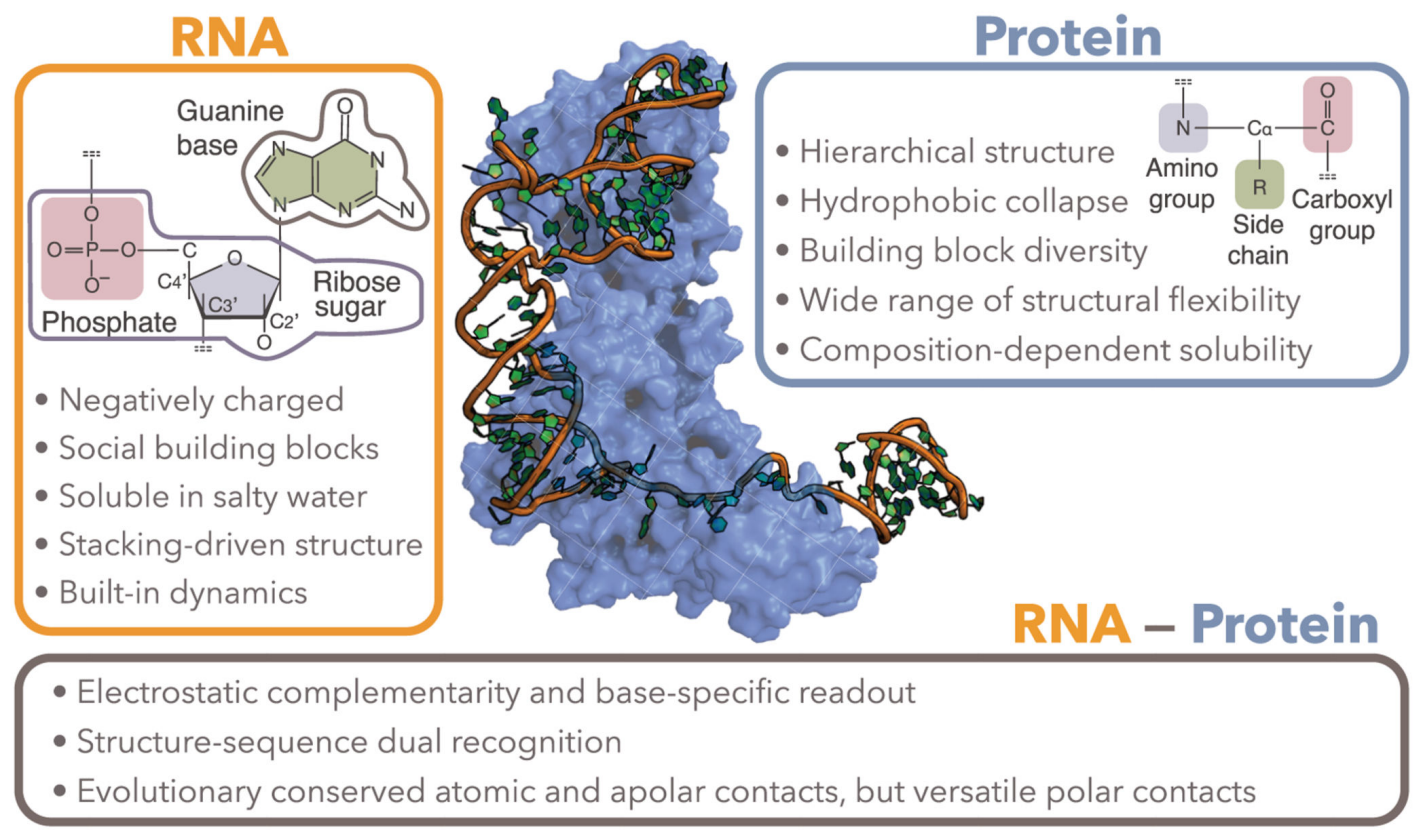
Example of a protein-RNA complex. We chose the cryo-electron microscopy structure of the human TUT1:U6 snRNA complex (PDB code: 9J8P [37]) to illustrate specific properties of RNAs, proteins, and their interfaces. This complex is essential for the efficiency of pre-mRNA splicing.

To overcome the scarcity of experimental data for protein-NA complexes, researchers have sought to leverage knowledge transfer from the more abundantly characterised protein and protein–protein complex structures. Nevertheless, nucleic acids display specific properties that distinguish them from proteins (Figure 2).

First, while proteins’ amino acid composition strongly influences their physicochemical properties, 3D geometry and solubility, nucleic acids exhibit a more hierarchical structural organization. Base composition primarily determines the secondary structure (2D base-pairing patterns), which in turn largely constrains the overall 3D fold. Second, the phosphate backbone is highly negatively charged, and works in concert with base stacking interactions to drive NA folding and stability. RNA molecules, specifically, are highly soluble in salty water and highly dynamic in solution. Their structure and dynamics often critically depend on the valence and ionic strength of the solution [16]. Third, the backbone of nucleic acids is much more flexible than the protein backbone, with 6 rotatable bonds per nucleotide versus only 2 per amino acid, which greatly increases the size of their conformational space. In particular, this allows RNA molecules, which often contain single-stranded (unpaired) nucleotides, to switch between multiple 3D conformations [17,18], thereby contributing to their functional diversity [19]. Consequently, RNA 3D structures are inherently more flexible and context-dependent than protein structures. This flexibility poses significant challenges while simultaneously offering opportunities for computational modelling: while it complicates direct 3D structure prediction, it highlights the importance of ensemble-based approaches and the value of secondary structure as a stable foundation.

The challenge of flexibility is most pronounced for modelling complexes containing single-stranded (ss) regions of RNA, such as those mediated by ssRNA-binding motifs [20], or those involving RNA aptamers, short fully single-stranded oligonucleotides that can bind proteins with high affinity and specificity. RoseTTa-FoldNA could obtain a correct model of the interface for only 1 out of 7 such test cases, and the authors highlighted the high flexibility of ssRNA as a major limitation [8]. Additionally, the induced-fit effect of proteins generates ssRNA conformations that differ from those experimentally observed in free ssRNA [21], contributing to the structural data scarcity challenge. This issue has driven the development of specific methods to build ssRNA conformations directly on the protein surface, based on fragment docking and assembly approaches [22–24].

### Evolutionary conservation of functional interactions

Much of the success of current protein structure prediction methods stems from their ability to capture amino acid covariations across homologous protein sequences, which reveal evolutionary constraints for maintaining their 3D structures. Likewise, nucleic acid sequence divergence patterns encode information relevant to their structures, and many covariation statistics, such as mutual information, G-test measures, and direct coupling analysis (based on the Potts model) have been explored for identifying conserved RNA structural contacts. These methods estimate counts or frequencies of nucleotide co-occurrences between pairs of positions from an input MSA — we refer readers to Ref. [38] for detailed mathematical formulations and comparative analysis. However, they face specific challenges in RNA analysis. Evolutionary pressures often act on base-pairing patterns rather than individual positions, and conserved RNA structures can still exhibit important differences across species [38]. Moreover, the degree to which RNA MSAs can inform us about RNA structure depends strongly on the type of RNA, with structurally relevant signals in messenger RNAs often confounded by the codon organisation patterns [39]. Additionally, erroneously including pseudogenes in ribozyme or ribosomal RNA alignments can destroy the covariation signals [38]. These difficulties, together with the paucity and low quality of RNA sequence data, may introduce biases and limitations in structural prediction methods relying on MSAs [40]. This has prompted efforts to develop improved automated and standardised tools for RNA sequence search, alignment and quality assessment [41].

In protein-RNA complexes, strongly covarying pairs have been identified within the protein interface [42] or the RNA interface [38]. However, identifying evolutionary pairwise couplings directly between interacting nucleic acids and amino acids remains difficult. The requirement for large coupled alignments limits the applicability of this strategy to a few bacterial complex families [43] and yields only slightly better than random predictive performance when benchmarked against dozens of complexes [44]. Despite these limitations in detecting direct evolutionary couplings, systematic analyses of protein-NA interface conservation have revealed important patterns. Functional NA binding sites at protein surfaces show distinct conservation profiles that correlate with their biological roles [45], while detailed analysis of protein-RNA interfaces has uncovered conserved contact patterns encompassing both geometric and chemical features [46]. Distance-based and apolar contacts in protein-RNA were found strongly conserved even between structural homologs sharing less than 20 % sequence identity, with non-conserved contacts representing a lower proportion than in protein–protein interfaces [46]. Such findings can inform which interaction patterns are effectively transferable between remote structural homologs by deep learning methods.

### Inverting the problem: designing RNA for known proteins

Beyond 3D structure prediction, the *de novo* design of idealised biomolecular shapes can reveal new insights about physical and structural constraints that might remain hidden when merely analysing natural proteins and nucleic acids [47]. Improving our ability to engineer functional protein-NA complexes can thus improve our understanding of their sequence-structure relationship. In recent years, several deep learning architectures have been developed to address this challenge (Figure 1b, d and Table 2).

Moving one step forward from simply designing RNA sequences that fold into a specific target 3D structure, the CARD method guides the design with knowledge about an interacting protein [26]. Specifically, CARD first encodes the target RNA structure with a Geometric Vector Perceptron Graph Neural Network (GVP-GNN) [48], which ensures SE(3)-equivariance, and then enhances this representation by attending to interacting protein residues converted into embeddings with a pre-trained protein LM. It achieves a higher recovery rate and macro F1 compared to inverse design methods that do not condition on a bound protein [26]. Taking this concept further, a few pioneering works have explored protein-conditioned co-design of NA sequence and structure [22,27,33,49], where the NA structure is not predetermined but emerges from the binding requirements to the target protein (Figure 1b,d, left panels). RNAFlow [27] leverages the flexibility of flow matching [50] to perform this task. Like CARD, it uses GVP-GNN to encode the input protein structure and a noisy version of the RNA bound to it, and then auto-regressively decodes an RNA sequence. The designed sequence is folded using RoseTTAFold2NA [8], which effectively serves as a denoiser and enables joint sequence and structure supervision. The method optionally exploits 3D conformations interpolated during flow matching, a strategy achieving high sequence recovery in motif scaffolding design tasks even at distant positions. Nonetheless, structural accuracy remains low. Recent developments have proposed energy-based iterative refinement with explicit bio-physical constraints to improve stereochemical quality, albeit with modest success [33].

Alternative approaches avoid dealing with the complexities of RNA 3D structure and instead focus on generating sequences conditioned on varying levels of protein structural data (Figure 1b,d, middle panels). The RNA Bidirectional Anchored Generation (BAnG) [28] introduces novel anchor tokens, representing the putative RNA binding site, from which it autoregressively generates the RNA sequence in both directions. To cope with the limited protein-RNA complex structural data in the PDB, RNA-BAnG leverages data augmentation by including DNA sequences and through a warm-up sequence reconstruction-only training phase over RNAcentral [51]. As a result, it enables out-of-the-box RNA sequence generation for any protein with a known or predicted structure. In contrast, RNAtranslator avoids relying on any structural information altogether [29]. It reframes protein-conditional RNA design as a sequence-to-sequence natural language translation problem: it translates an input protein sequence into a novel RNA binding sequence in an end-to-end fashion (Figure 1b,d, right panels). RNAtranslator is pre-trained on millions of experimental and predicted protein-RNA interacting pairs from the RNAInter Database [52], and then fine-tuned on experimentally validated pairs.

Paralleling the development of these design-oriented architectures, some works have aimed at re-purposing state-of-the-art biomolecular structure predictors methods for hallucination-based binder design [53,25] (Figure 1a, in purple). Recent improvements of the optimisation objective have been showcased through the design of aptamers that bind to the green fluorescent protein [25]. These promising results constitute a first step toward general-purpose biomolecular design frameworks.

### Integrating high-throughput omics data

The exciting recent advances in profiling interactions between proteins and nucleic acids [54] have generated large volumes of protein-NA interaction data through both *in vitro* and *in vivo* experimental methods. High-throughput omics approaches, including evolutionary selection methods (SELEX), direct binding assays (RNAcompete, RNA Bind-n-Seq), and *in vivo* cross-linking experiments (CLIP-Seq), provide RNA binding motif MSAs that can enrich and complement available structural information [55,56].

Currently, most deep learning methods exploiting protein-RNA omics data aim to model sequence-level binding preferences, without attempting to predict or incorporate structural information. One group of models is trained directly on experimental RNA sequences, including AptaDiff [34], which uses a discrete diffusion process to model SELEX-derived data [57] (Table 2). The diffusion process is conditioned on a latent representation learned via a variational autoencoder (VAE) with a hidden Markov model decoder [58]. Other approaches are not trained from scratch but instead fine-tune pre-trained foundation or general-purpose models using experimental binding data, adapting general representations to specific protein-RNA contexts. For instance, GenerRNA [35] is an RNA language model based on the GPT-2 architecture [59] pre-trained on RNAcentral data [51] and fine-tuned on RNAcompete and CLIP datasets. It utilises byte-pair encoding (BPE) tokenisation to compress the input sequences, at the expense of resolution. A second example is given by RNAGenesis [36], which is first pre-trained on large RNA sequence collections, including RNAcentral and the non-coding RNA subset of Ensembl [60], and then fine-tuned on a SELEX-derived dataset. RNAGenesis enhances the classical encoder-decoder transformer architecture by mapping the embeddings computed by the encoder into fixed-length latent vectors subjected to continuous denoising diffusion before decoding the output sequences. Moreover, it achieves both high compactness and high resolution through a hybrid n-gram tokenization scheme coupled with 1D Convolutional Neural Networks (CNNs) of varying kernel sizes. While these methods typically do not contribute to predicting protein-RNA structures themselves, attention scores between nucleic and amino acids may reflect binding interfaces [61].

Finally, some models incorporate feedback from other predictors to guide or refine the learning process, combining sequence-based learning with additional insights [62–65]. For instance, FAFormer [65] enables aptamer screening using structure encoding with E(3)-equivariant frame-averaged transformers, exploiting protein and RNA 3D models generated with AlphaFold and RoseTTAFoldNA, respectively.

### Conclusions and future directions

Despite significant advances in deep learning architectures for biomolecular structure prediction, substantial challenges remain in protein-NA structure prediction. Current state-of-the-art methods show limited accuracy when predicting structures outside their training distributions, particularly for RNA aptamers and novel conformations. While architectural innovations continue to emerge, our literature review and our own experience suggest that the choice of specific encoder and decoder designs may contribute less to predictive success than initially anticipated, with most frameworks demonstrating broadly comparable performance.

The primary determinants of model performance appear to be data quality and task formulation rather than architectural complexity alone. Protein-RNA interaction modeling benefits significantly from high-throughput omics data, which provide training opportunities that remain scarce for other systems such as protein-peptide interactions. However, the field would benefit from more robust, standardised experimental baselines for evaluating predictive performance. Current evaluation frameworks for protein-conditioned RNA design rely heavily on computational predictions, such as protein-RNA affinities estimated with deep learning scoring models.

#### Toward better splitting strategies and benchmarking

Deep learning approaches are particularly sensitive to data scarcity and quality issues. For protein-ligand interactions, strategies maximising training data diversity and quality while minimising task-specific leakage have proven valuable for boosting the performance of diffusion-based models [66]. Likewise, we envision that initiatives aiming at establishing comprehensive benchmarks for RNA structure and function modeling could lead to substantial progress. Several recent initiatives have begun to address this need [67,15,32]. RNAGLIB [67] for instance offers seven tasks (with datasets and splits) and a deep learning library for facilitating RNA 3D structure-based modelling. RNAGym [15] assesses the zero-shot performance of 19 baselines on three core tasks. For protein-RNA prediction, it offers a medium-size test set (127 complexes) and quantifies how much performance depends on similarity to the training set and correlates with coevolutionary pairwise couplings inferred with a statistical physics approach. The benchmark will benefit from including more baselines as it is currently limited to AlphaFold3 and RosettaFold2NA for this task. Ludaic and Elofsson [32] additionally evaluated Boltz-1 and HelixFold3 on a few tens of complexes. They showed that prediction accuracy increases with similarity to motifs found in the training set for all methods, confirming that training set similarity remains a critical factor.

#### Emerging patterns from language models

The technological breakthroughs achieved recently in self-supervised learning of massive amounts of unlabelled data hold promise for uncovering functional and structural signals in biological sequences [68–70]. Specifically, DNA language models trained to reconstruct genomic sequences at scale are effective in capturing inter-nucleotide dependencies representing RNA pseudoknots and tertiary structure contacts [70]. These models offer several key advantages over traditional methods. Firstly, by inputting single sequences, they provide a means to overcome the scarcity of high-quality MSAs for RNA molecules. The deep learning-powered docking method ProRNA3D-single [71] for instance, which combines protein and RNA sequence embeddings with geometric attention, recovers more native contacts than alignment-based state-of-the-art predictors in the low depth regime. Secondly, LM’s independence from known 3D templates makes them amenable to discovering previously unidentified binding modes and interaction patterns, as evidenced by the fact that some of the true positive contacts they capture are lost upon supervised fine-tuning [70]. Still, one pressing challenge in this direction consists of being able to capture the structural and functional interactions between very distant genomic regions. Indeed, nucleic acid sequences require much wider context compared to the protein ones. And while several architectures are now able to handle very long NA sequences as input, the signal resolution still deteriorates over 100 000 base pairs [72,73].

## Figures and Tables

**Table 1:** Deep learning approaches for protein-NA complex prediction. We list a selection of methods discussed in this review and indicate whether each method was evaluated in an independent benchmark. The term “Ludaic-Elofsson” refers to the Ludaic and Elofsson benchmark [32].

Method	Benchmark	Architecture	Strengths	Weaknesses
AlphaFold3 [10]	CASP, RNAGym, Ludaic-Elofsson	MSA-conditioned standard diffusion with transformer	broad molecular context	Memorization
RoseTTAFold2NA [8]	CASP, RNAGym, Ludaic-Elofsson	MSA-based, 3-track network for tokens, geometry, and coordinates, with SE(3)-transformer	Extended to broad molecular context in RoseTTAFold-all-Atom [30]	Poor modeling of local basepair network
HelixFold3 [31]	CASP, as part of Elofsson method [14], Ludaic-Elofsson	Adapted from AlphaFold3	broad molecular context	Does not outperform AlphaFold3
Boltz series [11, 12]	Ludaic-Elofsson	Adapted from AlphaFold3	broad molecular context, additional developments for affinity predictions	Does not outperform AlphaFold3
DeepProtNA [14]	CASP	Adapted from AF2, combines MSA with LM embeddings and secondary structure prediction	Used in several top performing predictors in CASP	Not published nor available

**Table 2:** Deep learning approaches for protein-conditioned RNA design. We list a selection of methods discussed in this review.

Method	Architecture	Strengths	Weaknesses
**From RNA struct and protein seq & struct, design RNA seq**			
CARD [26]	GVP-GNN enhanced with pLM	Improves recovery and macro F1	Limited availability of training and
	embeddings, transformer self-attention and decoder		evaluation data
**From protein seq & struct, co-design RNA seq & struct**			
RNAFlow [27]	Noise-to-sequence GVP-GNN stacked with RF2NA within flow-matching framework	can condition on multiple interpolated conformations, high sequence recovery far from binding motif	Low structural accuracy
RNA-EFM [33]	Adapted from RNAFlow, refinement with biophysical energy constraints	Explicit account of stereochemical realism	Low structural accuracy
**From protein seq & struct, design RNA seq only**			
RNA-BAnG [28]	bidirectional autoregressive generation, geometric attention (IPA) from AF2	Alleviates the need for binding site definition while keeping it as model constraint	No experimental validation of the predictions
**From protein seq only, design RNA seq only**			
RNAtranslator [29]	Classical encoder-decoder transformer	Avoids dealing with geometry, generalises to novel or synthetic proteins	No experimental validation of the predictions, limited scalability
**Leveraging HTS data and large corpus of RNA seq to design RNA seq**			
AptaDiff [34]	VAE trained on SELEX libraries, discrete diffusion conditioned on the latent space	Leverages motif dependent-embeddings	Lack of end-to-end training
GenerRNA [35]	Decoder-only transformer, fine-tuned on CLIP data	General-purpose LM	Low resolution, no experimental validation of the predictions
RNAGenesis [36]	Transformer enhanced with latent diffusion, fine-tuned on SELEX data	General-purpose LM, both compact and high-resolution	Does not yet explicitly models DNA or proteins

## Data Availability

No data was used for the research described in the article.
